# Comparative effect of intraduodenal and intrajejunal glucose infusion on the gut–incretin axis response in healthy males

**DOI:** 10.1038/nutd.2015.6

**Published:** 2015-05-18

**Authors:** T Wu, S S Thazhath, C S Marathe, M J Bound, K L Jones, M Horowitz, C K Rayner

**Affiliations:** 1 Discipline of Medicine, The University of Adelaide, Royal Adelaide Hospital, Adelaide, South Australia, Australia; 2 Centre of Research Excellence in Translating Nutritional Science to Good Health, The University of Adelaide, Adelaide, South Australia, Australia

## Abstract

The region of enteral nutrient exposure may be an important determinant of postprandial incretin hormone secretion and blood glucose homoeostasis. We compared responses of plasma glucagon-like peptide-1 (GLP-1), glucose-dependent insulinotropic polypeptide (GIP), insulin and glucagon, and blood glucose to a standardised glucose infusion into the proximal jejunum and duodenum in healthy humans. Ten healthy males were evaluated during a standardised glucose infusion (2 kcal min^−1^ over 120 min) into the proximal jejunum (50 cm post pylorus) and were compared with another 10 healthy males matched for ethnicity, age and body mass index who received an identical glucose infusion into the duodenum (12 cm post pylorus). Blood was sampled frequently for measurements of blood glucose and plasma hormones. Plasma GLP-1, GIP and insulin responses, as well as the insulin:glucose ratio and the insulinogenic index 1 (IGI_1_) were greater (*P*<0.05 for each) after intrajejunal (i.j.) than intraduodenal glucose infusion, without a significant difference in blood glucose or plasma glucagon. Pooled analyses revealed direct relationships between IGI_1_ and the responses of GLP-1 and GIP (*r*=0.48 and 0.56, respectively, *P*<0.05 each), and between glucagon and GLP-1 (*r*=0.70, *P*<0.001). In conclusion, i.j. glucose elicits greater incretin hormone and insulin secretion than intraduodenal glucose in healthy humans, suggesting regional specificity of the gut–incretin axis.

## Introduction

Roux-en-Y gastric bypass leads to remarkable improvements in glycaemic control in type 2 diabetes, associated with an enhanced incretin effect (the phenomenon of an amplified insulin response to enteral vs intravenous (i.v.) glucose, mediated by glucagon-like peptide-1 (GLP-1) and glucose-dependent insulinotropic polypeptide (GIP)).^1, 2^ This may relate to diversion of nutrients more distally in the small intestine, as similar benefits are observed with the endoluminal sleeve device.^3^ We hypothesised that bypassing the duodenum would elicit a greater response of the gut–incretin axis to small intestinal glucose infusion, and compared plasma GLP-1, GIP, insulin and glucagon, and blood glucose responses to a standardised glucose infusion into the proximal jejunum and duodenum in healthy humans.

## Subjects and methods

Ten healthy males received an intrajejunal (i.j.) glucose infusion; data regarding blood glucose, and plasma insulin, glucagon and GLP-1 have been reported previously.^4^ These, together with plasma GIP, were compared with 10 healthy males who received an intraduodenal (i.d.) glucose infusion (Table 1). All subjects provided written, informed consent. Protocols were approved by the Royal Adelaide Hospital Human Research Ethics Committee.

On the evening before each study (~1900 hours), each subject consumed a standardised beef lasagne meal (McCain, Wendouree, VIC, Australia), and then fasted from solids and refrained from liquids after 2200 hours. Subjects attended the laboratory at ~0800 hours the following day, when a multilumen silicone catheter (Dentsleeve International, Ontario, Canada) was positioned transnasally in either the duodenum or proximal jejunum (infusion port: 12 vs 50 cm beyond the pylorus) by peristalsis, with monitoring of antral and duodenal transmucosal potential difference.^4^ In the i.j. study, a balloon was inflated 30 cm beyond the pylorus to exclude the duodenum.^4^ Enteral glucose was then infused at 2 kcal min^−1^ for 120 min (*t*=0–120 min). An i.v. cannula was inserted into a forearm vein for blood sampling. Blood samples were collected at frequent intervals into ice-chilled EDTA tubes and immediately centrifuged at 3200 r.p.m., for 15 min at 4 °C. Plasma was separated and stored at −70 °C until analysed.

Blood glucose was measured by glucometer (Medisense Precision QID, Bedford, MA, USA). Plasma total GLP-1 was measured by radioimmunoassay (GLP1T-36HK; Linco Research, St Charles, MO, USA) with a sensitivity of 3 pmol l^−1^, and intra- and inter-assay coefficients of variation (CVs) of 6.8% and 8.5%, respectively. Plasma total GIP was measured by radioimmunoassay modified from a previously published method,^5^ with a sensitivity of 2 pmol l^−1^, and intra- and inter-assay CVs of 5.1% and 8.8%, respectively. Plasma insulin was measured by enzyme-linked immunosorbent assay (10-1113; Mercodia, Uppsala, Sweden) with a sensitivity of 1 mU l^−1^ and intra- and inter-assay CVs of 2.7% and 7.8%, respectively. Plasma glucagon was measured by radioimmunoassay (GL-32 K; Millipore, Billerica, MA, USA) with a sensitivity of 20 pg ml^−1^, and intra- and inter-assay CVs of 15% and 10.5%, respectively.

Student's unpaired *t*-test was used to compare subject demographics, fasting biochemical measures and insulinogenic index 1 (IGI_1_), which was calculated from insulin (I) and glucose (G) concentrations, as (I_30_-I_0_)/(G_30_-G_0_), to evaluate β-cell responsiveness.^6^ Two-way analysis of variance, with treatment and time as factors, was used to compare responses between the two studies. Pearson's correlation was used to assess relationships between integrated area under the curve (ΔAUC), calculated using the trapezoidal rule, for incretin hormones and both IGI_1_ and ΔAUC for glucagon. Analyses were performed using Prism 6.0 software (GraphPad, La Jolla, CA, USA). Data are represented as mean±s.e.; *P*<0.05 (two sided) was considered statistically significant.

## Results

**Figure 1:**
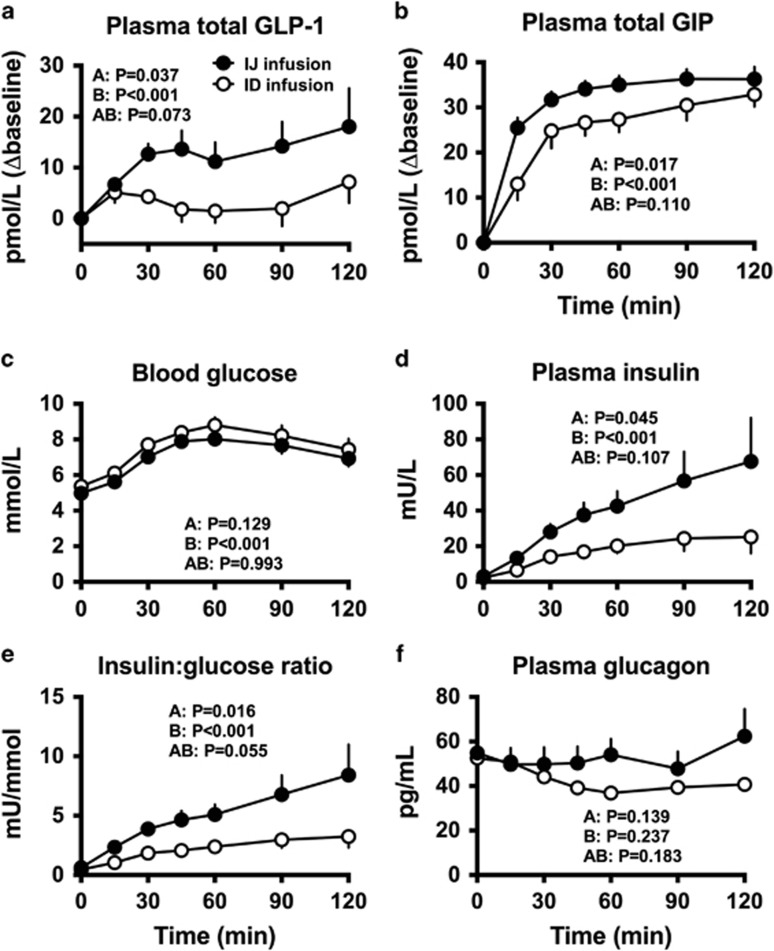
Comparative responses of plasma total GLP-1 (**a**), total GIP (**b**), blood glucose (**c**), plasma insulin (**d**), the insulin:glucose ratio (**e**) and plasma glucagon (**f**) during an intrajejunal (i.j.) and intraduodenal (i.d.) glucose infusion at the rate of 2 kcal min^−1^ (*t*=0–120 min) in healthy males (*n*=10 each). *P*-values are for (A): differences by experiment, (B) differences over time and (AB): differences due to the interaction of experiment and time. Data are represented as mean±s.e.; *P*<0.05 was considered statistically significant.

Demographics and fasting values did not differ between the two studies (Table 1). During enteral glucose infusion, plasma GLP-1 increased substantially with i.j. administration (time effect: *P*<0.001), but minimally with i.d. delivery (time effect: *P*=0.003), and was greater for i.j. than i.d. glucose (treatment effect: *P*=0.037). Plasma GIP increased promptly on both days (time effect: *P*<0.001), and concentrations were also greater with i.j. glucose (treatment effect: *P*=0.017). Blood glucose concentrations increased to ~8 mmol l^−1^ on both days, and were numerically, but not significantly, lower with i.j. glucose. However, plasma insulin, the insulin:glucose ratio and IGI_1_ (12.0±1.4 vs 5.6±1.3 mU mmol^−1^) were all greater with i.j. glucose (treatment effect: *P*<0.05 for all). Plasma glucagon did not change with i.j. glucose, but fell slightly with i.d. glucose (time effect: *P*<0.001), without significant difference between the two (Figure 1).

On pooling data from all 20 subjects, IGI_1_ was related directly to ΔAUC for total GLP-1 and GIP (*r*=0.48, *P*=0.036 and *r*=0.56, *P*=0.012, respectively). ΔAUC for glucagon was related directly to ΔAUC for GLP-1 (*r*=0.70, *P*<0.001), but not GIP.

## Conclusion

We showed that i.j. glucose elicited greater incretin and insulin release than i.d. glucose in healthy males, supporting the concept that directing nutrients more distally in the small intestine could ameliorate type 2 diabetes. Blood glucose did not differ significantly, probably because of the modest glycaemic excursion in these healthy individuals. However, we cannot rule out a type 2 error due to the small size of each group.

Enteral glucose was delivered at 2 kcal min^−1^ on both days, which is within the physiological range of gastric emptying.^7^ The infusion site was 38 cm more distal in the i.j. study; given that the small intestine can absorb glucose at 2 kcal min^−1^ per 30 cm in health,^8, 9^ this would have allowed substantially greater interaction of glucose with more distal gut regions where GLP-1-releasing L-cells are more abundant. This is consistent with observations of enhanced GLP-1 secretion after implantation of a duodenal-jejunal sleeve.^3^ Alternatively, exclusion of the duodenum may have a role in ameliorating diabetes (the ‘foregut hypothesis').^10^ The relative contribution of more distal gut exposure vs duodenal exclusion should be evaluated in subsequent studies. The greater GIP response to i.j. glucose may also reflect a higher density of GIP secreting K-cells in the proximal jejunum than duodenum in humans, as seen in pigs.^11^ Moreover, the expression of sodium glucose co-transporter-1 may be of relevance^12^—this was reported to be greater in the jejunum than duodenum in rodents.^13^

Plasma glucagon decreased during i.d. glucose infusion, but remained unchanged during i.j. glucose infusion. This may be partly accounted for by the interplay between GLP-1 and GIP on pancreatic α-cells; the glucagonostatic effect of GLP-1 is blunted,^14^ and the glucagonotropic effect of GIP is potentiated,^15^ in the context of relatively low blood glucose concentrations. Surprisingly, a direct relationship between glucagon and GLP-1 was observed, which might imply a contribution of GLP-2, a hormone co-secreted with GLP-1 and potent at stimulating glucagon.^16^ Differences in glucagon between the i.d. and i.j. studies may have contributed to the lack of difference in blood glucose concentrations.

We inflated a balloon in the i.j. study to exclude the duodenum; this *per se* would be unlikely to enhance incretin secretion.^17^ Exclusion of bile in the i.j. study would not affect GIP secretion,^18^ and if anything would favour a reduced GLP-1 response.^4^ In the i.d. study, it is possible that some of the infused glucose could have refluxed into the stomach, but this should have been minimised by the increased pyloric tone associated with i.d. glucose infusion.^19^

Our study has limitations, which should be recognised. First, our observations were made in a parallel study design, with a relatively small number of subjects in each group; however, the subjects were well matched and the differences in plasma incretin hormones and insulin were consistent between the two studies. Therefore, increasing the sample size in a crossover study is unlikely to alter the study conclusions. Furthermore, small intestinal glucose was delivered into two sites at a single rate. It would be of interest to employ different rates of glucose infusion into various sites in order better to characterise the regional specificity of the gut–incretin axis. Finally, the balance of evidence seems to suggest alterations in secretion and/or action of incretin hormones in obesity and type 2 diabetes.^20^ For example, the secretion of GLP-1 is reportedly impaired in obesity, while GIP secretion may be enhanced.^20^ In the case of type 2 diabetes, the insulinotropic effect of GIP is largely diminished, although that of GLP-1 is better preserved. These pathophysiological features warrant further evaluation of the gut–incretin physiology in the presence of obesity and/or type 2 diabetes.

In summary, our observations indicate that i.j. glucose elicits greater incretin hormone and insulin secretion than i.d. glucose in healthy humans, suggesting regional specificity of the gut–incretin axis.

## Figures and Tables

**Table 1:** Demographics and fasting biochemical measures of subjects in the intrajejunal (i.j.) vs intraduodenal (i.d.) study^a^

	*i.j. study*	*i.d. study*
Subjects	10 healthy males (8 Caucasians and 2 Asians)	10 healthy males (8 Caucasians and 2 Asians)
Age (years)	33.4±6.0	33.4±5.3
BMI (kg m^−2^)	24.5±1.1	25.0±1.0
Fasting glucose (mmol l^−1^)	5.0±0.1	5.4±0.1
Fasting insulin (mU l^−1^)	3.1±0.4	2.5±0.5
Fasting insulin:glucose ratio (mU mmol^−1^)	0.6±0.1	0.5±0.1
Fasting GLP-1 (pmol l^−1^)	19.2±2.0	24.1±2.4
Fasting GIP (pmol l^−1^)	14.0±1.6	15.3±2.7
Fasting glucagon (pg ml^−1^)	54.9±7.9	52.5±2.6

Abbreviations: BMI, body mass index; GIP, glucose-dependent insulinotropic polypeptide; GLP-1, glucagon-like peptide-1.

a Data are represented as mean±s.e.; Student's unpaired *t*-test was used to determine the statistical significance. *P*<0.05 was considered statistically significant.

## Notes

The study was supported by the National Health and Medical Research Council (NHMRC) project grant (No. APP1066815). TW has received research funding from AstraZeneca. CKR has received research funding from Merck, Eli Lilly and Novartis. MH has participated in the advisory boards and/or symposia for Novo Nordisk, Sanofi, Novartis, Eli Lilly, Merck Sharp & Dohme, Boehringer Ingelheim and AstraZeneca, and has received honoraria for his activity. KLJ's salary is provided by an NHMRC Senior Career Development Award (627011). The remaining authors declare no conflict of interest.
